# Corals Like It Waxed: Paraffin-Based Antifouling Technology Enhances Coral Spat Survival

**DOI:** 10.1371/journal.pone.0087545

**Published:** 2014-01-28

**Authors:** Jan Tebben, James R. Guest, Tsai M. Sin, Peter D. Steinberg, Tilmann Harder

**Affiliations:** 1 Centre for Marine Bio-Innovation, School of Biological, Earth and Environmental Sciences, University of New South Wales, Sydney, New South Wales, Australia; 2 Advanced Environmental Biotechnology Centre, Nanyang Technological University, Singapore, Singapore; Environmental Monitoring, Informatics and Dynamics, Tropical Marine Science Institute, National University of Singapore, Singapore, Singapore; Leibniz Center for Tropical Marine Ecology, Germany

## Abstract

The early post-settlement stage is the most sensitive during the life history of reef building corals. However, few studies have examined the factors that influence coral mortality during this period. Here, the impact of fouling on the survival of newly settled coral spat of *Acropora millepora* was investigated by manipulating the extent of fouling cover on settlement tiles using non-toxic, wax antifouling coatings. Survival of spat on coated tiles was double that on control tiles. Moreover, there was a significant negative correlation between percentage cover of fouling and spat survival across all tiles types, suggesting that fouling in direct proximity to settled corals has detrimental effects on early post-settlement survival. While previous studies have shown that increased fouling negatively affects coral larval settlement and health of juvenile and adult corals, to the best of our knowledge, this is the first study to show a direct relationship between fouling and early post-settlement survival for a broadcast spawning scleractinian coral. The negative effects of fouling on this sensitive life history stage may become more pronounced in the future as coastal eutrophication increases. Our results further suggest that targeted seeding of coral spat on artificial surfaces in combination with fouling control could prove useful to improve the efficiency of sexual reproduction-based coral propagation for reef rehabilitation.

## Introduction

The early post-settlement stage of corals, i.e. the first few weeks and months after pelagic larvae settle and metamorphose, is generally considered a critical life history stage.Newly settled individuals are very vulnerable and early post-settlement mortality is spatially, temporally and taxonomically variable [1], [2]. Most reef building corals exhibit typical type III survival curves [3] with high early mortality and increased probability of survival with increasing age and/or size [4]–[6]. The extent of mortality during the early post-settlement period can strongly influence the abundance and distribution of adult populations [7]. Understanding the factors that influence mortality during this period is therefore important to the effective management and rehabilitation of coral reefs.

Ecological studies of early post-settlement mortality of corals are difficult because corals less than one year of age (defined as coral recruits) are usually too small (i.e. <1 cm diameter) to be identified *in situ*. Therefore, most field studies of early population dynamics omit newly recruited corals, focusing instead on juvenile corals that can be seen with the naked eye, typically >1 cm diameter [8]. Corals of this size are likely to be at least one year old, and consequently our understanding of the factors that influence the mortality bottleneck in the first year of a coral's life remains limited [7].

Studies of early coral mortality have for the most part relied on monitoring survival of recently settled corals *in situ* or *ex situ* on artificial substrata. Experiments with laboratory-reared coral spat transplanted to the reef have shown that survival of newly settled corals (days to a few weeks after settlement) is typically <15% in the first three to four months [4], [6], [9], [10]. However, direct evidence for the causes of high coral spat mortality is limited and often constrained by infrequent sampling [6], [11]–[13]. Known causes of mortality of coral spat include smothering [14], sedimentation [15], chemical warfare [16]–[18], pathogens [19], [20], settlement on unsuitable substrates [21], [22], and accidental removal by grazing fish or direct predation [9], [23].

Fouling is the accumulation of micro- (bacteria, fungi, protozoa, etc.) and macroorganisms (algae and animals) on immersed hard substrata. The interaction between corals and fouling organisms, particularly filamentous algae, is generally detrimental for the health of adult corals and settlement of coral larvae [24]–[26]. The extent and type of benthic fouling is likely to affect mortality during the early life stages of corals [27]. Fouling can impact coral spat in at least two ways: through competition from adjacent fouling organisms on the substratum or by direct fouling of the spat themselves by microorganisms or motile propagules of other macroorganisms [14]. Furthermore, the microtopography provided by fouling may enhance passive deposition of inanimate material such as sediment onto coral recruits [28] or provide a refuge for microbial pathogens [19]. For example, in a common Hawaiian coral, mortality of planulae and settled spat increased in the presence of macroalgae and possibly resulted from increased microbial activity [29].

As well as being important for our understanding of natural coral mortality, knowing the impact of fouling on early post-settlement survival may also have important implications for active rehabilitation of reefs. Coral reefs are in decline in many areas of the world [30], [31] and active rehabilitation efforts are taking place globally [32]. Such efforts typically focus on propagation and transplantation of corals produced by asexual fragmentation [33], [34]. More recently, sexual reproduction of corals has been used to propagate large numbers of coral larvae for enhancing recruitment on small areas of degraded reefs [35] or for settlement and rearing on artificial substrata until corals are large enough to be transplanted [36]–[38]. Control of fouling may significantly enhance the success of both approaches, but in particular that of sexual propagation techniques, because newly settled coral spat are more vulnerable to mortality due to negative interactions with fouling organisms.

This study investigated the impact of fouling on the early post-settlement survival of coral spat of the scleractinian coral *Acropora millepora*. Fouling was manipulated with non-toxic antifouling coatings to modulate the extent of fouling on substrata colonised by laboratory-reared corals. Our objectives were to directly investigate the impact of fouling on spat survival, and to work towards a technology that could overcome the bottleneck of spat mortality for reef rehabilitation techniques that rely on sexual reproduction.

## Results

### Settlement on different tile materials

**Figure 1:**
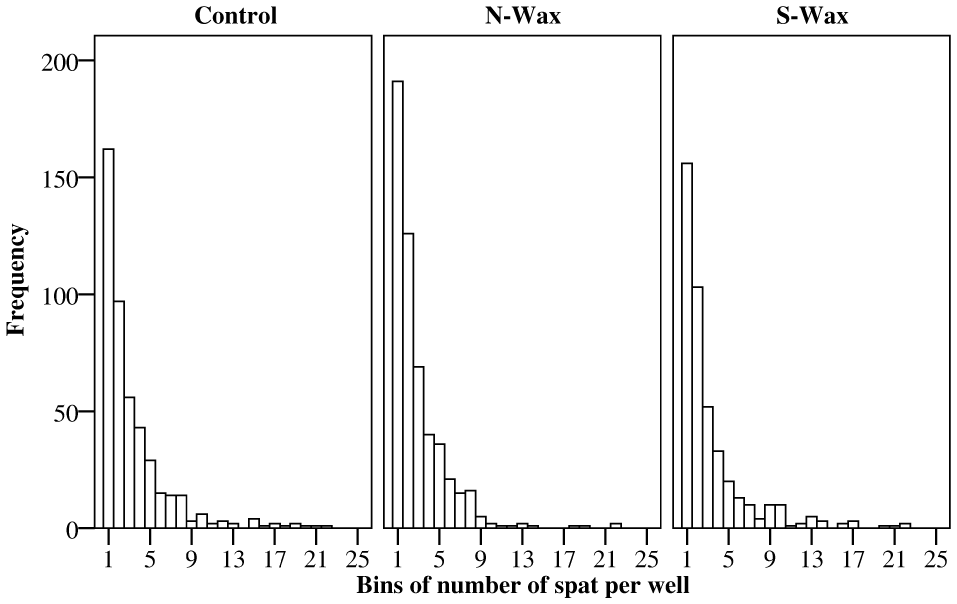
Frequency distributions of the number of coral spat per well for each treatment. The x axis shows the number of coral spat in each well, the y axis shows the frequency of wells containing different numbers of spat for all replicate tiles. The three graphs compare frequency distributions for the three tile treatments (terracotta control, N-Wax, S-Wax).

Initial densities of *Acropora millepora* spat settling on experimental settlement tiles were analysed by one-way ANOVA. This served to ensure that spat densities on different tile materials were the same in order to allow accurate survival analyses (Table 1). There were no significant differences among the three tile materials (uncoated terracotta and terracotta with antifouling waxes) at the beginning of the experiment in terms of the total number of spat per tile (*F*_2, 45_ = 0.14, *P* = 0.86) and the proportion of wells containing at least one living spat per tile (*F*_2, 45_ = 1.06, *P* = 0.35). More than one third of the settlement wells contained a single coral spat for all treatments and more than two thirds of wells had fewer than three coral spat (Figure 1). Further, there was no significant difference of variance of spat numbers per well among treatments, indicating that the distribution of single and multiple spat per well did not differ among treatments (*F*_2,45_ = 0.1, *P* = 0.9) (Figure 1).

**Table 1:** Mean and standard error of settled coral spat per well for number of wells containing at least one spat per tile for each tile type.

	Teracotta control	N-Wax	S-Wax
Average number of spat per well (mean ±SE)	1.96±0.007	1.98±0.007	1.82±0.007
Average number of wells with ≥1 spat per tile (mean ±SE)	29±2.9	33±2.6	27±3.0
Total number of spat	1541	1558	1322
Total wells with ≥1 spat	459	529	439

Total number of spat and number of wells containing at least one spat for each tile type is also shown.

### Spat survival on different surfaces

**Figure 2:**
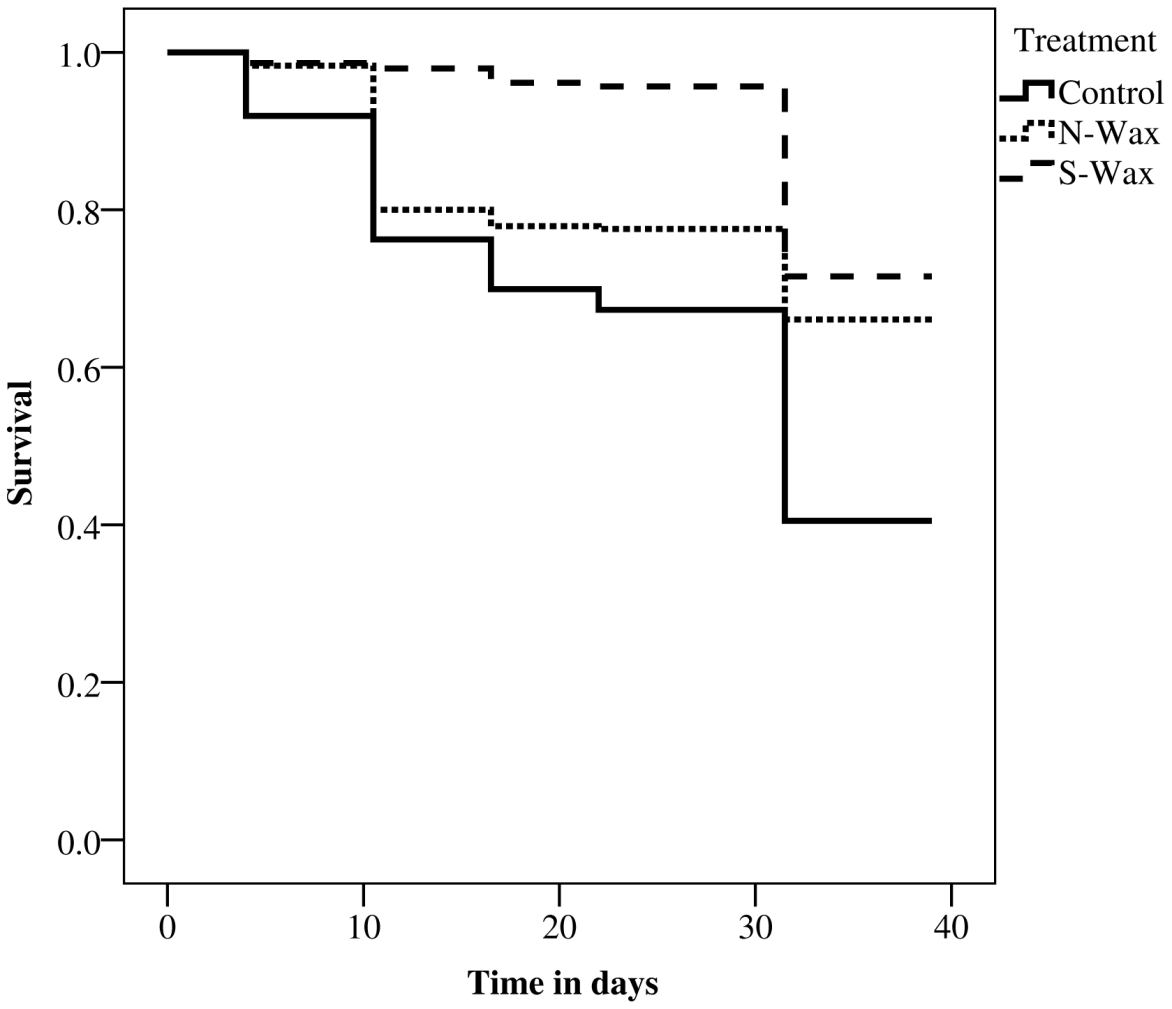
Comparison of survival curves for *Acropora millepora* spat on tiles with modulated fouling cover. Kaplan-Meier estimated survival is shown pooled over each tile type (terracotta control, N-Wax; S-Wax) over a time of 39 days.

Comparison of the Kaplan-Meier survival curves over 39 days revealed a mean survival time of 27.9±0.6 days (mean ± SE) for coral spat on untreated terracotta tiles. This was significantly lower (log-rank test, *P*<0.001) than survival times on both antifouling coatings (31.8±0.5 d for the N-Wax, 36.8±0.2 d for the S-Wax), whereas no significant difference in survival times between the wax treatments was observed (Figure 2). At the end of the experiment (39 days), the majority of coral spat across all treatments had taken up zooxanthellae and many had started to bud daughter polyps, indicating normal development. After 39 days, the proportion of wells containing at least one live coral spat was 40.4±9.1% (mean ± SE, n = 460) for untreated terracotta tiles, 67.2±5.6% (mean ± SE, n = 529) for N-Wax tiles and 72.3±4.1% (mean ± SE, n = 439) for S-Wax tiles. There was a significant effect of the tile type on percentage survival after 39 days (two-way ANOVA, *F*_2,6_ = 7.96, *P* = 0.021), no significant interaction between tile type and tank (*F*_6,36_ = 0.78, *P* = 0.59) and no significant effect of the tank (*F*_3,36_ = 0.45, *P* = 0.72) (Table 2). Survival on N-Wax (Tukey HSD, *P* = 0.025) and S-Wax (*P* = 0.007) tiles was significantly greater than on control tiles but did not differ between each wax tile treatment (*P*<0.05). The experimental procedure did not bias any particular treatment in terms of spat quantities, densities and frequencies of spat per well and tile. However, to ensure density related survival was not dependent on the tile material, separate survival analysis was carried out for wells containing single and multiple spat separately. The mean survival times of those spat starting as single polyps (control 24.7±1.1 d; 31.7±0.5 d for the N-Wax; 36.0±0.3 d for the S-Wax; log-rank test, P<0.001) did not differ in comparison to wells with multiple spat and to all wells combined. Thus, it is unlikely that there was any density related survival dependent on the tile material.

**Table 2:** Results of two-factor ANOVA for percent coral spat survivorship.

	df	MS	F	P
Tile type (S)	2	0.4681	7.96	*
Tank location (T)	3	0.0339	0.45	NS
S x T	6	0.0588	0.59	NS
Residual	36	0.0759		
Total	47			

* = P<0.05, NS = not significant.

### Fouling cover

**Figure 3:**
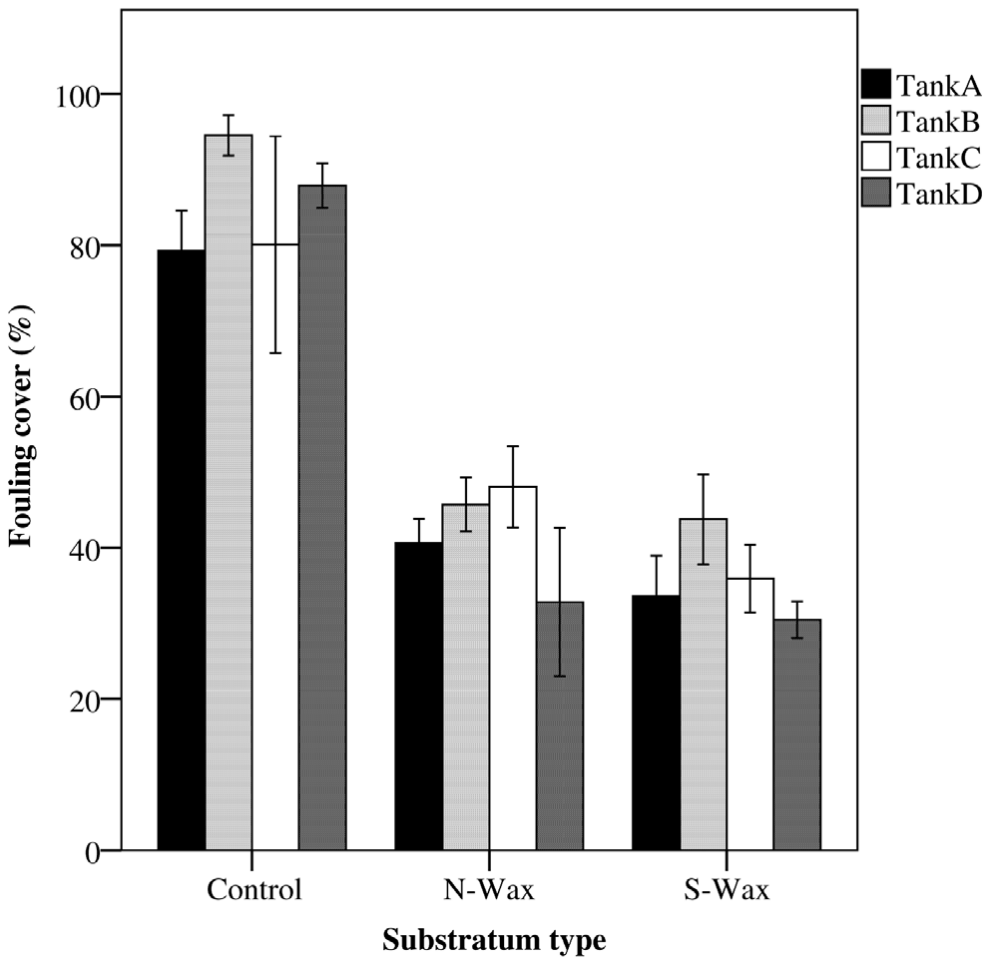
Comparison of fouling cover for among treatments. Average percentage cover of fouling (mean +/− se) is shown for each experimental tank and for each treatment (terracotta control, N-Wax, S-Wax).

**Figure 4:**
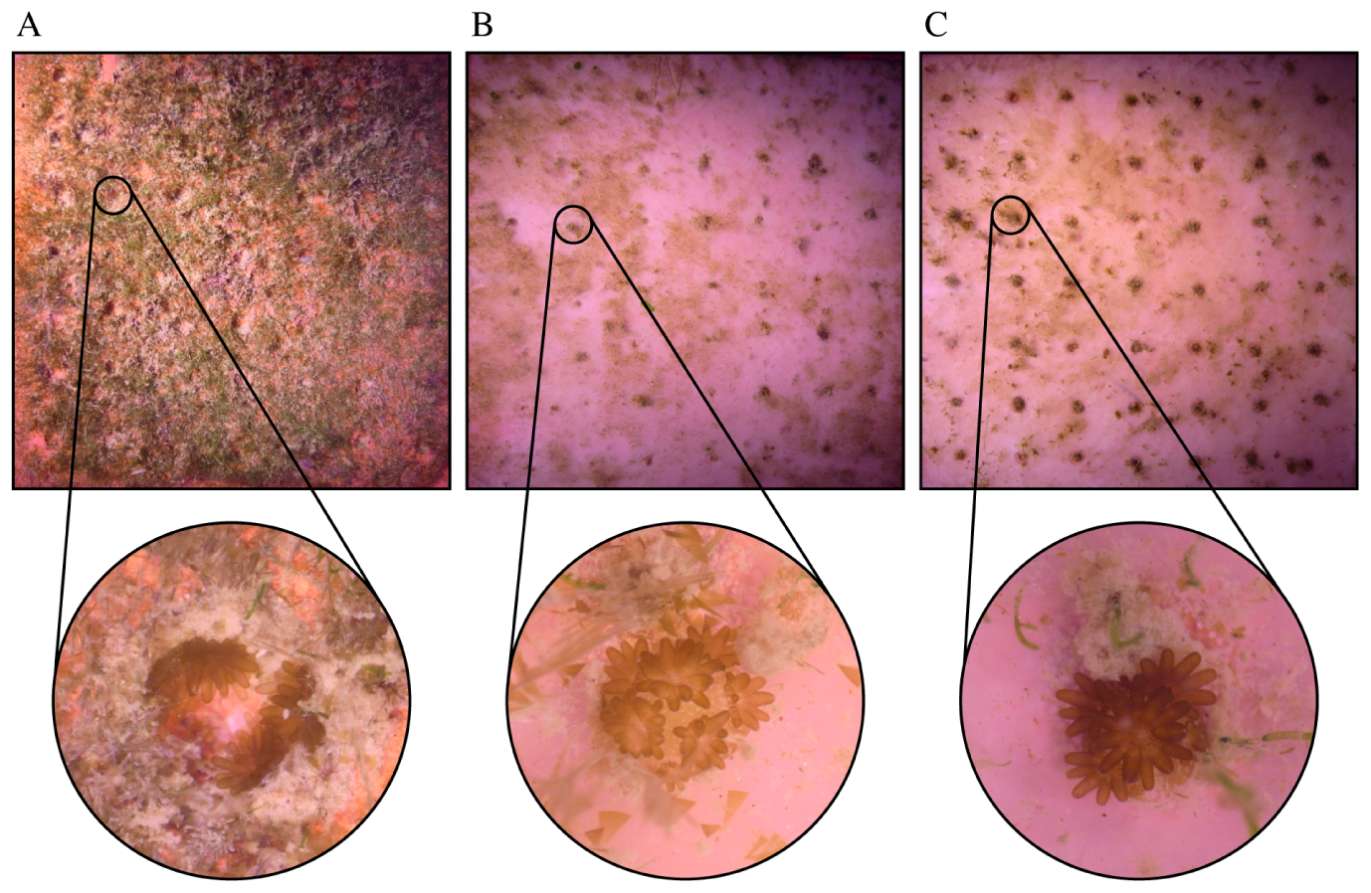
Representative pictures of fouling cover on the three tile types. Square pictures show the entire settlement tile (20×20 cm). Round inserts show microscope images of spat in the settlement wells (diameter ∼10 mm) for A: terracotta control; B: N-Wax; C: S-Wax after 39 days.

Percentage cover of fouling was significantly higher on control tiles (84.9±3.6%, mean ±SE) than on the N-Wax tiles (41.3%±3.2%, mean ± SE, Tukey HSD, *P*<0.0001) and S-Wax tiles (35.2±2.5%, mean ± SE, Tukey HSD, *P*<0.0001), with no significant difference between the two wax types (Figure 3, Table 3). Representative pictures of fouling cover on the three tile types are shown in Figure 4. There was no effect of the tank location (two-way AVOVA, *F*_3,36_ = 2, *P* = 0.12) nor of the interaction between tank location and tile type (two-way AVOVA, F_6,36_ = 71.3, *P* = 0.80).

**Table 3:** Results of two-factor ANOVA for fouling on tiles.

	df	MS	F	P
Tile type (S)	2	48.93	75.29	**
Tank location (T)	3	1.6494	2	NS
S x T	6	0.6499	0.79	NS
Residual	36	0.8262		
Total	47			

Data were square root transformed prior to analysis. * = P<0.05, NS = not significant.

### Spat survival versus fouling

**Figure 5:**
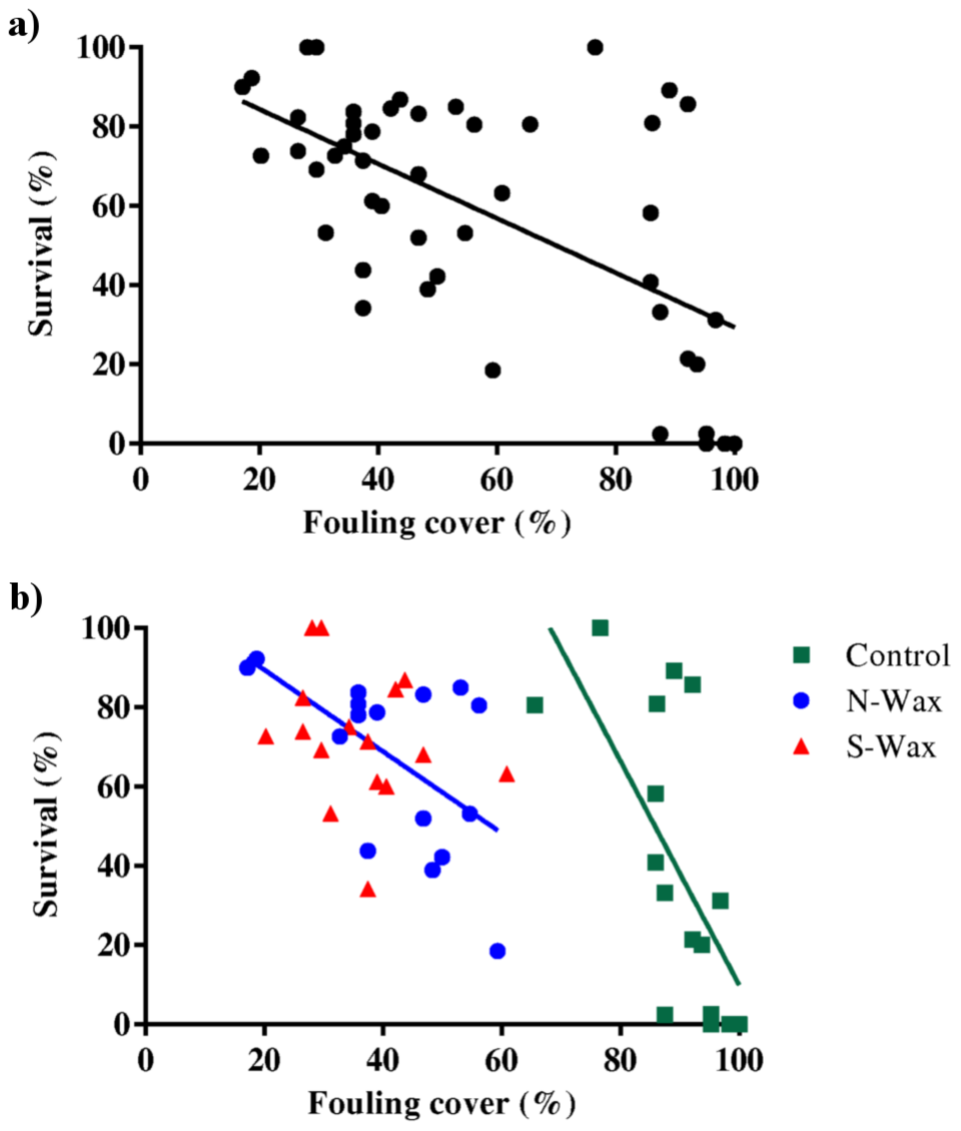
Comparison of the relationship between survival and fouling cover. Graph shows a scatter plot of percentage survival after 39 days over percentage fouling cover a) pooled over all tile types and b) each of the three treatments individually (terracotta control, N-Wax; S-Wax). Results of the regression analysis are shown in Table 4.

Regression analysis of the relationship between the proportion of coral spat survival and percentage fouling cover on all tiles pooled (Figure 5 a, Table 4) indicated a significant negative influence of fouling on survival (N = 48; F = 27.06; *P*<0.001, R^2^ = 0.37). Separate analyses of spat survival on each tile type indicated significant negative effects for control (N = 16; F = 11.24; *P*<0.005; R^2^ = 0.45) and N-wax treatments (N = 16; F = 6.97; *P* 0.02; R^2^ = 0.33), but there was no significant correlation between spat survival and fouling cover for the S-wax treatment (N = 16; F = 0.98; *P* = 0.34) (Figure 5 b, Table 4).

**Table 4:** Linear regression analyses for survival vs. fouling cover (Figures 5 a, b).

	N	F	Significance	R^2^
Pooled	47	27.06	<0.001	0.37
Control	15	11.24	<0.005	0.45
N-Wax	15	6.97	0.02	0.33
S-Wax	15	0.98	0.34 (NS)	

NS = not significant.

## Discussion

During the early post-settlement stage, coral spat face a major mortality bottleneck. However, the natural rates of mortality and the factors affecting mortality of newly settled spat are poorly understood. In this study we investigated the impact of fouling on survival of newly settled coral spat of *Acropora millepora ex situ* by manipulating fouling cover with antifouling coatings. These experiments provide one of the few data sets on early post-settlement mortality of corals (also see [29]), and demonstrate that benthic fouling has a significant impact on the extent of early post-settlement mortality for a scleractinian coral. Our results also suggest that the combined manipulation of larval settlement (via settlement inducers) and fouling (via antifouling coatings) may be useful for sexual reproduction based rehabilitation efforts.

The *ex situ* experimental approach taken in the present study allowed us to control and quantify the extent of fouling on settlement substrata and examine its effect on early post-settlement mortality in the absence of other confounding factors such as grazing, predation and sedimentation. Traditionally, examinations of the early life stages of corals have involved random settlement of coral spat on biologically conditioned substrata. This approach has disadvantages for tracking and quantifying survival because the coral spat settlement, is spatially heterogeneous with spat tending to aggregate, fuse and settle on cryptic surfaces of settlement substrata (e.g. edges of tiles). In this study we controlled the spatial distribution of settlement using natural cues, resulting in comparable settlement among treatments and replicates. In addition, the use of environmentally benign antifouling coatings was successful in significantly reducing fouling cover on experimental tiles compared to control tiles without the need for the disruptive removal of fouling organisms.

Both antifouling treatments significantly reduced fouling cover in comparison with the control, and survival of coral spat on the antifouling treatments was significantly greater than that of spat on untreated control tiles. Fouling cover significantly negatively correlated with spat survival when pooled across all tile types and for spat on control and N-wax tiles (though not on silicon wax tiles). The combined data of average percent survival and fouling cover per tile showed a significant linear relationship between fouling and survival for both pooled data as well as control and N-wax individually.

The wax-based antifouling coatings used in this study are non-toxic (food-grade) and initial settlement was the same across different tile types. This observation suggests that the primary effect of the different tile types on survivorship was likely due to the modulation of fouling cover. We speculate that the effect of fouling is especially relevant for coral spat, because these early life stages lack many of the defence strategies present in older, more established coral colonies (e.g. sweeper tentacles [39], [40]). Although the wax coating with silicone showed both a significant reduction in fouling (lowest of all treatments) and increase in survival (highest of all treatments), there was no apparent linear relationship between these factors, suggesting that the silicone additive had an effect on spat survival independent of the variation in (macro)fouling cover. While there was a clear link between fouling cover and mortality in the control treatment, there were a number of tiles with both high survival and high fouling cover (see Figure 5 b). A possible explanation for this observation is that the composition of the fouling community, as well as overall cover, determines the extent of post-settlement mortality. We speculate that the silicone additive in the wax not just altered the fouling cover but also the composition of the fouling community. Further studies are clearly needed to examine the effect of different fouling communities on juvenile coral health, the precise mechanism that causes coral mortality (e.g. shading, allelopathy, microbial shifts etc.) and the role that different anti-fouling coatings play in modulating the fouling community.

In this study, fouling was quantified as total cover without further qualifying the fouling community. However, we observed the macrofouling community to be almost entirely comprised of filamentous microalgae and detritus. The interaction between algae and corals is of profound importance to the health of coral reefs [41], [42]. The antagonistic effects of algae on corals range from direct allelopathy [43] to the possible harbouring of pathogens or disruption of coral microbial communities [20], [44], which can inhibit coral recruitment [26], [45]–[47] and lower the survival of juvenile and adult corals [24]–[26]. Survival of planulae of the coral *Montipora capitata* is significantly reduced in the presence of macroalgae, especially when the corals settled on the surface of these algae [29]. The results from another field study indicate that a reduction in competition with other sessile epibiota can increase survival of settled hard coral spat [27].

The results of the present study suggest that antagonistic algal–coral interactions may also affect the survival of coral spat during the early post-settlement stage. While the precise mechanisms that lead to increased mortality of coral spat remain unclear, macrofouling may indirectly influence coral spat survival by harbouring microorganisms or by weakening the coral's resistance to microbial infections [29].

Many degraded coral reefs are characterised by an increased abundance of non reef-building benthic fouling organisms, such as fleshy macroalgae and heterotrophic filter feeders [48]. Tropical reefs worldwide have already undergone a phase shift from coral- to fleshy algal-dominated cover [49]–[52]. As coastal eutrophication increases globally [53], interactions between fouling organisms and corals are likely to become more pronounced and frequent. In the context of this increased fouling pressure on already degraded reefs, the results of this study may have broad implications for reef rehabilitation. Coral spat reared and settled *ex situ* for subsequent nursery rearing generally have very low survival rates [36], rendering the costs of these techniques prohibitively high. The application of antifouling technologies can potentially improve the efficiency and economic feasibility of sexual reproduction-based coral propagation by increasing the yield of coral surviving through vulnerable early life history stages. Future studies would benefit from identifying the fouling-organisms associated with high coral spat mortality rates to facilitate the design of specific antifouling technologies that counteract these organisms. Further research is also required to assess whether these techniques can prove effective in the field and can therefore be used in other rehabilitation efforts, for example by reducing the amount of manual cleaning required during nursery rearing of asexually propagated corals [54].

## Methods

### Ethics statement

All work, including collection of corals, was done with permission of the Singapore National Parks Board under a joint project between Nanyang Technological University and Singapore National Parks Board project entitled “Rehabilitating Singapore's Reefs Using Sexually Reared Corals” (Project number: M4060980). All colonies were returned to the reef within a week after spawning and reattached to minimize damage to the reef.

### Spawning and culturing of corals

The major annual peak in coral spawning in Singapore occurs during the week after the full moon of March or April [55]. Five colonies of the scleractinian coral *Acropora millepora* were collected on the day of the full moon (7 April 2012) from Kusu Island (1° 13′ 31.9″ N, 103° 51′ 29.2″ E). The colonies were transferred to the Tropical Marine Science Institute (TMSI) at St John's Island and held in outdoor tanks (1800 l) with sand-filtered flow through seawater (SFSW). The colonies were isolated each night 2 h prior to the predicted spawning time (21:00 h) and kept isolated until 23:00 h or until spawning occurred. All five colonies spawned on nights 1, 2 and 3 after the full moon (8 to 10 April) between 21:00 h and 22:30 h. Larval culture techniques followed Heyward and Negri [56]. Gamete bundles were scooped from the water surface immediately after spawning. Sperm and eggs were separated by pouring gamete bundles through a submerged 100 µm mesh sieve into a 20 l bucket containing UV-sterilised, 0.2 µm filtered sea water (UV-FSW). Sperm and eggs from three colonies respectively were fertilised in two separate 20 l buckets. After 1 h the fertilised embryos were removed by surface scooping, washed three times in 50 l of UV-FSW and transferred to three larval rearing tanks (500 l) with continuous flow-through of UV-FSW, where they were maintained at low densities (≤0.5 larva ml^−1^) until they were competent to settle. Settlement competency of the larvae was monitored daily with larval bioassays using crustose coralline algae (CCA, ca. 5 mm^2^, uncharacterised species) in sterile six-well culture plates at 28–30°C. Larvae were settled on tiles when 80% of the larvae settled in response to CCA (6 d post spawning).

### Preparation of settlement tiles

Uncoated terracotta tiles (controls) and two terracotta tile treatments with wax anti-fouling coatings (20×20 cm in size, n = 16 of each type) were prepared as settlement substrata. Paraffin waxes are environmentally benign (the waxes used here are food-grade), and wax coating is one of the oldest strategies to reduce fouling and epibiotic coverage on immersed artificial structures (since 300 BC [57]). Fouling was manipulated with wax antifouling coatings in preference to techniques that might be detrimental to coral spat survival, for example, manual removal or toxic anti-fouling paints.

Terracotta tiles were primed with resin (EZ100050P, Ecozean Pty Ltd, Sydney, Australia) to increase wax adhesion and dip-coated with wax (EZ001-2, Ecozean Pty Ltd, Sydney Australia), hereafter referred to as N-Wax. The same wax was used in a second treatment with an addition of 0.1% silicone oil (hereafter referred to as S-Wax), as addition of silicone affects the antifouling or foul release capacity [58]–[60].

For the main survival study, control tiles and tiles coated with wax were prepared as follows: 49 wells (5 mm wide and 2 mm deep, 7×7 arrays) were drilled into each tile to provide a settlement site for coral larvae. Each well was then filled with 1 µl of powdered CCA in sterile FSW to render the wells inductive to larval settlement. Since the remaining tile area was not biologically conditioned, the aim of this method was to selectively settle coral larvae within the wells. Batches of tiles (n = 5 per treatment) containing wells filled with CCA were placed into tanks with competent coral larvae (from 6 d post spawning, density <0.5 larvae ml^−1^, 500 l tank volume) for no longer than 24 h. Tiles with settled spat were kept in FSW until the start of the experiment to ensure that all spat were exposed to the onset of fouling at the same time point. This procedure took 5 d and resulted in 48 tiles (16 per treatment) containing at least 10 wells with coral spat. Less than 10% of the total number of coral larvae settled on tile surfaces outside the designated wells. These spat were ignored for the purpose of the survival study in order to maintain consistency and to facilitate monitoring of survival.

### Survival studies

Tiles were placed flat and fully immersed in four shallow (9 cm deep) tray tanks (120 l) with constant flow-through sand filtered sea-water. Four tiles per treatment were fully interspersed within each of the four tanks (n = 12 tiles per tank). The tanks were cleaned immediately before the tiles were introduced to create similar conditions in each tank. Subsequently, the tanks developed a fouling community of macroalgae (brown, green, red and CCA), microalgae, ascidians, polychaetes and anemones. The tanks were not cleaned during the experiment to allow persistent fouling pressure on the experimental tiles. Coral spat survival was scored at the beginning of the experiment followed by weekly intervals over a course of 39 d.The tiles were gently removed from the tanks, placed into seawater trays and spat survival in microwells was visually determined under the microscope. Survival was scored on a per well basis, with wells scored as alive when there was at least one living polyp in a well. This was done to avoid problems associated with discriminating individual coral spat, as they tend and bud daughter polyps and may aggregate with fusion common among individuals that settle close together. This procedure did not bias any particular treatment in either spat quantities, densities or frequencies of spat per well and tile (Figure 1, Table 1).

### Quantification of surface fouling on experimental tiles

The mean percentage of fouling cover on experimental tile surfaces was measured after 39 d in 5×5 mm quadrats positioned directly over three randomly selected microwells per replicate tile. Fouling was estimated via photographs of each quadrat and analysed using the Coral Point Count Software with Excel extensions (CPCe) [61]. Thirty-two points per 25 mm^2^ quadrat were analysed using a stratified random point count methodology for four columns by four rows and two points per cell. The surfaces were analysed according to two categories: fouled (e.g. macroalgae, microalgae, sediment) and non-fouled (non-fouled coral spat and tile surface with no visible fouling). While analysis of the species composition of the fouling community would have been desirable, this was not possible without disturbing the integrity of the fouling community.

### Statistical analyses

The survival functions of spat over 39 d on three different tile types was estimated using the Kaplan–Meier method [62].Survival functions among tile treatments were compared (control, N-Wax and S-Wax tiles) using log-rank tests. Since non-parametric survival analysis cannot incorporate replication, data from all tanks were pooled for this analysis. To examine for tank x treatment interactions, differences in percentage survival of coral spat and percentage cover of fouling organisms per replicate tile after 39 d were analysed by two-way ANOVA with tile type as a fixed factor (three levels) and tank as a random factor, orthogonal to tile type (four levels). ANOVA was followed by post-hoc pair-wise comparisons among treatments using a post-hoc Tukey HSD test [63]. Cochran's C test was used to test for homogeneity of variances and where significant heterogeneity was found the data were square-root transformed prior to analysis by ANOVA. The relationship between the proportion of spat surviving after 39 d and percentage cover of fouling per tile was analysed by linear regression pooled across all tiles types (n = 48) and for each tile type individually (n = 16).
